# Analytical Framework for Identifying and Differentiating Recent Hitchhiking and Severe Bottleneck Effects from Multi-Locus DNA Sequence Data

**DOI:** 10.1371/journal.pone.0037588

**Published:** 2012-05-25

**Authors:** Ori Sargsyan

**Affiliations:** Theoretical Biology and Biophysics and Center for Nonlinear Studies, Los Alamos National Laboratory, Los Alamos, New Mexico, United States of America; University of Florence, Italy

## Abstract

Hitchhiking and severe bottleneck effects have impact on the dynamics of genetic diversity of a population by inducing homogenization at a single locus and at the genome-wide scale, respectively. As a result, identification and differentiation of the signatures of such events from DNA sequence data at a single locus is challenging. This paper develops an analytical framework for identifying and differentiating recent homogenization events at multiple neutral loci in low recombination regions. The dynamics of genetic diversity at a locus after a recent homogenization event is modeled according to the infinite-sites mutation model and the Wright-Fisher model of reproduction with constant population size. In this setting, I derive analytical expressions for the distribution, mean, and variance of the number of polymorphic sites in a random sample of DNA sequences from a locus affected by a recent homogenization event. Based on this framework, three likelihood-ratio based tests are presented for identifying and differentiating recent homogenization events at multiple loci. Lastly, I apply the framework to two data sets. First, I consider human DNA sequences from four non-coding loci on different chromosomes for inferring evolutionary history of modern human populations. The results suggest, in particular, that recent homogenization events at the loci are identifiable when the effective human population size is 50000 or greater in contrast to 10000, and the estimates of the recent homogenization events are agree with the “Out of Africa” hypothesis. Second, I use HIV DNA sequences from HIV-1-infected patients to infer the times of HIV seroconversions. The estimates are contrasted with other estimates derived as the mid-time point between the last HIV-negative and first HIV-positive screening tests. The results show that significant discrepancies can exist between the estimates.

## Introduction

Hitchhiking and severe bottleneck effects have similar signatures on the population genome by reseting the molecular clock. However, their impacts at the genome level are on different scales. The hitchhiking effect has a local signature because recombination breaks down linkage disequilibrium between sites on the genome; consequently, the locus completely linked to a site under a positive selection becomes homogenous in the population [1]. In contrast, after relatively quick recovery of a population from a severe bottleneck, it becomes genome-wide homogeneous. Identifying and differentiating recent such events at a single locus can be challenging because both processes have similar signature on the genetic diversity at single locus. Thus, multi-locus DNA sequence data can be a powerful source for this purpose.

After a recent homogenization event at a neutral locus, the accumulated genetic diversity at the locus and the elapsed time are positively correlated when assuming constant molecular clock. To quantify the relation between genetic diversity at the neutral locus in a low recombination region and the time elapsed since a recent homogenization event, Griffiths [2], Tajima [3], and Perliz and Stephan [4] used Wright-Fisher reproduction model with constant population size and infinite-sites model [5] for the dynamics of genetic diversity at the locus. They derived analytical expressions for the expected number of polymorphic sites in a sample of DNA sequences from such a locus. Although this framework is computationally efficient for inferring the elapsed time, it is applicable only for a single locus.

Simulation based inference methods have been developed for the same problem to include an exponential population growth model and full polymorphism data in samples of DNA sequences [6], [7]. Although such methods have flexibility to include more complex evolutionary scenarios, they are computationally more intense.

I consider the same setting as in [2], [3], and [4] to develop an analytical framework for identifying and differentiating recent homogenization events at multiple neutral loci in low recombination regions. The loci are considered to be evolving independently, for example, when the loci are on different chromosomes or on same chromosome but far apart. I derive an analytical expression for the probability distribution of the number of polymorphic sites in a sample of DNA sequences. Based on this, I described likelihood-ratio based tests for identifying and differentiating recent homogenization events at multiple loci. I apply the framework to two data sets. First, I use human DNA sequence data to infer evolutionary history and origin of modern human populations. Second, I use HIV DNA sequences sampled from HIV-1-infected patients to infer the times of HIV seroconversions.

## Methods

### The population genetic model

Genetic diversity at a neutral locus, in a low recombination region, affected by a recent homogenization event is a result of mutations accumulated at the locus since the homogenization event. To model the dynamics of genetic diversity at such a locus after the homogenization event, this paper combines the infinite-sites mutation model and the Wright-Fisher reproduction model with constant population size. The parameters in the model represent the effective population size 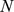, the elapsed time 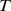 since the last homogenization event, mutation rate 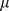 per generation per sequence, and the (effective) generation time 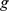.

Variation in a sample of DNA sequences drawn from a population evolving according to this model can be described as a combination of genealogical and mutation processes. The genealogical process traces ancestral lineages of the sample back in time until the recent homogenization event at time 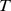 and stops earlier if the most recent common ancestor of the sample is more recent than the homogenization event. When 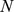 is large and the time in this process is measured in 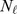 generations, the genealogical process can be approximated by a coalescent process derived from the standard coalescent [8]–[12]. Here 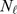 is a scaled population size at the locus, determined by 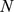 and the type of the chromosome on which the locus is located: 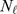 is equal to 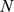 for the case of a haploid population; for a diploid population with 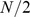 males and 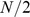 females, 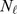 is equal to 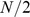, 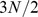, 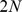, or 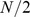 if the locus is on the 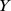, the X, the autosomal chromosome, or on the mitochondrial DNA, respectively. In this process the ancestral lineages of the sample are traced until time 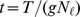 and mutations are added on the branches of the genealogy as independent Poisson processes with rates equal to 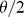, 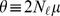. In the infinite-sites model, each mutation occurs at a nucleotide site that has not been mutated before.

## Results

### Probability distribution of the number of polymorphic sites in a sample of DNA sequences

Under the model described above, the probability distribution of the number of polymorphic sites 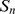 in a sample of 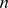 DNA sequences can be represented as
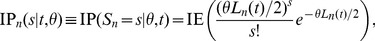
(1)where 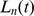 is the total length of the genealogy of 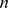 sequences. This equation suggests that the probability can be expressed through the derivatives of the moment generating function 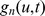 of 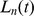, defined as 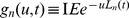:
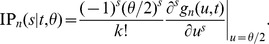
(2)


Griffiths [2] derived an analytical expression for 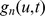, but it can not be easily used to derive expressions for the derivatives of 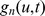. In the following lemma, I derive an expression for 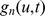, which allows easily to derive analytical expressions for the derivatives of 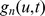. Note that this expression also allows to invert the moment generating function 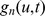 and to derive an analytic expression for the density function of 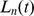. The latter result is presented in the lemma of the Text S1.

**Lemma 1** *The moment generating function* 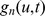 *can be represented as*

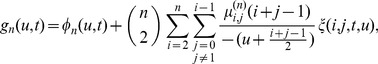
(3)
*where*

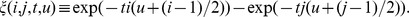

*The coefficients* 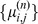 *are determined by the following recurrence relations with initial conditions:*

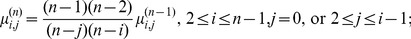
(4)

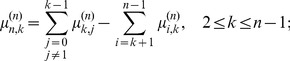
(5)

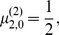
(6)

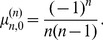
(7)


The prove of Lemma 1 is provided in the Text S1.

Note that the coefficients 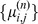 satisfy the following identities
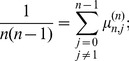
(8)

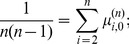
(9)

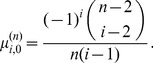
(10)The identities are used in the proof of Lemma 1, and also for identifying numerical instability issues with computation of 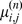 based on (4)–(7) when using decimal approximations instead of exact computations. The proof of the identities can be done by combining mathematical induction with (4)–(7), the details not shown.

**Figure 1:**
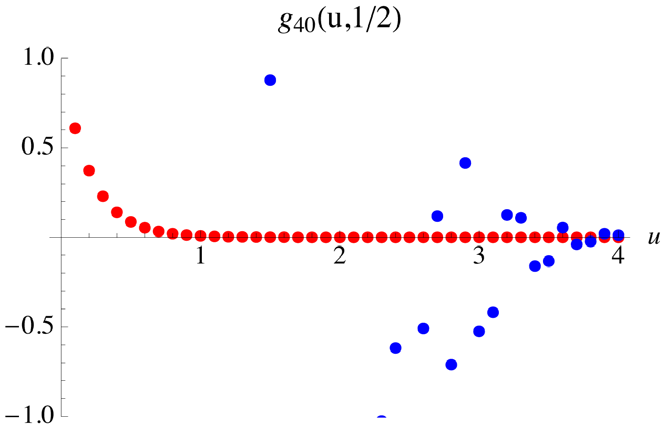
Illustration of numerical instability of the expression (3). The moment generating function 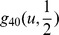 is plotted for the same range of values of 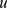 in red and blue dots by using the expressions (11) and (3), respectively. The numerical instability of the expression (3) is obvious because the values of 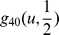 must be between 0 and 1 for any positive 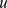.

Expression (3) is used to derive expressions for the derivatives of 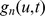, but for computational purposes they are modified to derive numerically stable expressions. The following procedure is applied to the expressions to solve the instability issue: for each 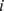, 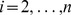, the terms with factor 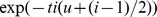 are combined together and the common term is factored out. For example, a numerically stable expression for 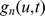 is
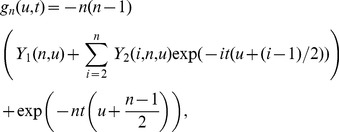
(11)where
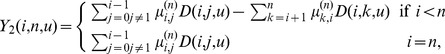


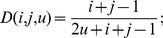


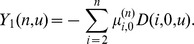
The numerical instability of the expression (3) is illustrated in Figure 1.

To derive a numerically stable expression for 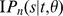 by using (2), first expressions are derived for the derivatives of 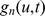 with respect to 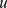 by using Lemma 1 and the identity
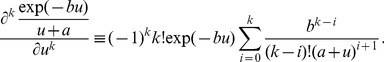
(12)After applying the numerical stabilization procedure (described above) to these expressions, a numerically stable expression for the probability distribution is
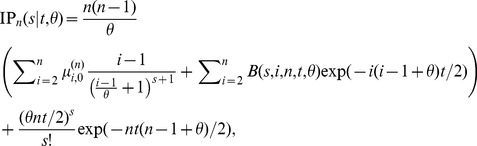
(13)where
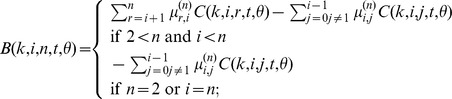


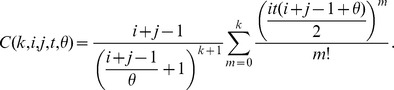
I have implemented the formula (13) and all the other formulas in this paper in a program written in *Mathematica* [13]. The program is used to carry out all the calculations and visualizations in this paper. The program uses *Mathematica*'s ability of doing exact computations with fractions, as a result avoiding numerical instability issues. The program is available from the author on request.

Note that when 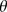 is large, the following approximation holds for the probability distribution 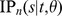:
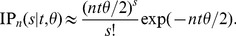
(14)The right side of the approximation corresponds to the probability distribution of the number of polymorphic sites in a sample of 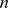 sequences under a “simple” model where the ancestral lineages of the sample are traced until time 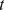 without coalescence. Note that population size 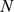 is not a factor in this model because the right side of the above approximation can be represented as
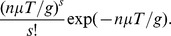



### Mean and variance of the number of polymorphic sites in a sample of DNA sequences

In previous studies [2]–[4], expressions have been derived for the mean number of polymorphic sites in a sample of DNA sequences from a locus affected by a recent homogenization event. An expression for the variance is also derived in [4], but this expression is implicit because it includes integral expressions. Using a similar approach as in the previous section, I derive a numerically stable expressions for computing the mean and variance of the number of polymorphic sites in a sample of DNA sequences from such a locus. The conditional probability distribution of 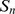 when 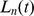 is given is Poisson with a mean of 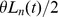. The mean and variance of 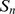 can be expressed as follows:
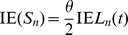
and
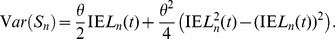
Expressions for the first and second moments of 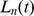 are derived by taking the first and second derivatives of (3) with respect to 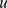 and evaluating them at 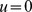. After applying the numerical stabilization procedure (described in the previous section) to these expressions, the first and second moments of 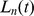 can be computed using the following formulas:
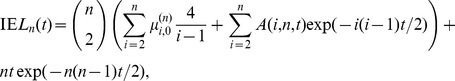
where 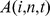 is defined as
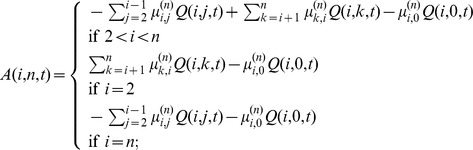


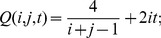


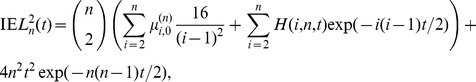
where 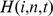 is
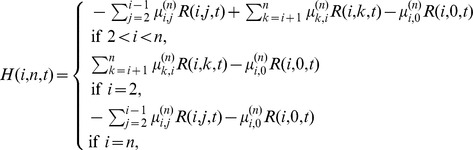


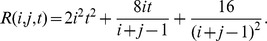



### Three tests for identifying and differentiating recent homogenization events at multiple loci

Using the probabilistic framework developed above, three likelihood-ratio based tests are considered in this section for identifying and differentiating recent homogenization events at independently evolving multiple neutral loci in low recombination regions.

#### Test I

To identify a recent homogenization event at a locus based on the number of polymorphic sites in a sample of DNA sequences, the hypothesis 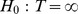 versus 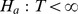 is considered. The null hypothesis 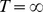 represents a case in which ancestral population was evolving according to the Wright-Fisher model with constant population size. The null hypothesis can be tested by defining minus twice of the log of the likelihood-ratio statistics as
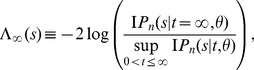
and comparing it with a 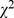 distribution with d.f.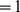.

Note that 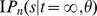 corresponds to the probability distribution of the number of polymorphic sites in a sample of DNA sequences when the genealogy of the sample is modeled by the standard coalescent and assumed the infinite-sites model for mutations. Tavaré [14] derived an expression for 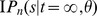, which also follows from (13) by taking 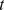 to 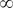:
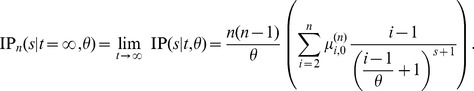
(15)


#### Test II

Suppose we know, for example, from other studies, that a recent homogenization event occurred at time 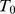 and we want to identify if this event had impact on a locus of interest. Symbolically, the following hypothesis can be stated
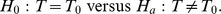
The null hypothesis can be tested by comparing minus twice of the likelihood-ratio statistics
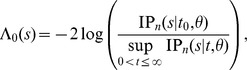
with a 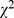 distribution with d.f.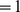, where 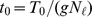.

Based on this approximation, for each 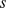 a 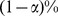 confidence interval
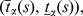
of 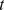 is determined by solving the equation
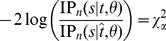
with respect to 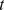; 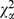 is the 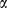 critical value of the 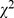 distribution. Note that when 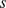 is 0, then 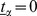 and 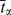 is the solution of the above equation. A 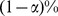 confidence interval of 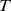 is 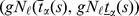. One can use a similar approach to estimate a 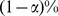 confidence interval for 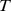 when inferring the elapsed time of a recent severe bottleneck event based on DNA sequence data from independently evolving multiple neutral loci. Another approach for this case is described below.

#### Test III

For a case of 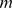 independent neutral loci, let the loci be labeled from 1 to 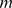, and 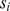 be the number of polymorphic sites in a sample of 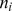 sequences at locus 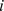, 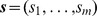. To test if the multiple loci are affected by the same recent homogenization event, the following hypothesis is considered:
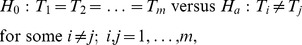
where 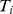 is the time elapsed since a recent homogenization event at locus 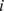. The null hypothesis can be tested by comparing the statistics
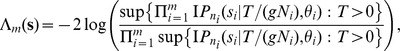
with a 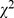 distribution with d.f.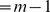, where 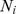 is the scaled population size at locus 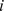; 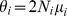 is the scaled mutation rate at locus 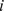, and 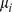 is the mutation rate per generation per sequence at locus 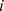.

### Inferring the time of a recent severe bottleneck event based on polymorphism data at multiple loci

The following steps can be taken to infer the time 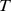 of a recent severe bottleneck event from DNA sequence data at independently evolving multiple neutral loci in low recombination regions. The likelihood function for such a data set can be computed as a product of likelihood functions from each locus by using formula (13). Thus, in case of 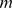 independent loci, and 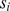 polymorphic sites in a sample of 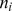 sequences at locus 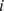, 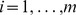, the maximum likelihood estimator 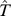 of 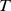 can be derived by solving the equation
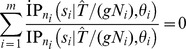
with respect to 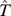, where 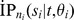 is the derivative of 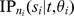 with respect to 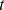. It is assumed that the scaled mutation rate 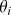 and the scaled population size 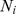 at locus 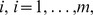 are known.

To estimate a 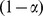
% confidence interval of 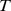, the Central Limit Theorem based approximation can be used when the following conditions hold: (1) The number 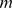 of the loci is large; (2) the loci are on same type of chromosomes (as a result 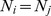); (3) samples of DNA sequences from each locus have the same size (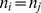); (4) the lengths of the sequences from the loci are equal 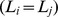. Thus, the 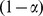
% confidence interval of 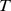 can be computed as
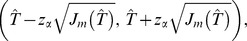
where 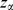 is the 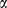 critical value from the standard normal distribution; 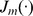 is observed Fisher information, which can be computed using the formula
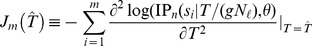


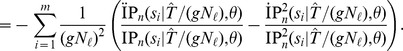
For evaluating the above expression, numerically stable expressions for the first and second derivatives of 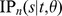 with respect to 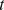 can be derived by using (13) and the numerical stabilization procedure.

### Application of the method for inferring recent homogenization events from human genome

Anthropological and archeological data strongly support “Out of Africa” hypothesis for the origin and evolutionary history of modern humans [15]–[19]. The hypothesis underlies two major events: *Homo sapiens* (ancestors of modern humans) emerged in Africa between 150,000 and 200,000 years ago (kya) and dispersed to other regions of the world sometimes before 50,000 years before present (yr B.P.). Studies based on mitochondrial and Y-chromosome support this hypothesis [20]–[28]. However, studies based on DNA sequence data from coding and non-coding loci on autosomal and X-chromosome show that the most recent common ancestor of 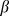-globin gene [29], the X chromosome gene for the pyruvate dehydrogenase E1 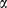-subunit [30], and the non-coding loci 22q11.2 [31], 17q23 [32], Xq13.3 [33] are much older than 200,000 yr B.P. These inferences are based on the framework of the standard coalescent, in which the effective human population size and the mutation rate per nucleotide site per generation are considered to be 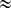
10000 [34] and 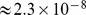 [35]–[37], respectively.

In contrast to this approach, I use the framework developed in this paper to analyze some of data sets used in the studies mentioned above. I apply the framework to DNA sequences from four non-coding loci (22q11.2, 17q23, Xq13.3, YAP) in low recombination regions on chromosomes 22, 17, X, and Y to identify and differentiate recent homogenization events associated with the “Out of Africa” hypothesis. The data sets are published in [25], [31]–[33], respectively, and their summary is in Table 1. First, I consider commonly accepted estimates for values of the parameters in the model: the effective human population size 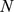 to be 10000; the mutation rate 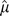 per nucleotide site per generation to be 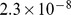; the human (effective) generation time 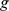 to be 20 years. Mutation rate 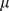 per generation per sequence at each locus is computed as 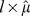, where 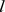 is the length of the DNA sequences at the locus. After applying Test I for this set of parameter values to each of the four data sets, the power of detecting a recent homogenization event at any of the loci is very weak (the 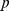-values close to 1, data not shown). In this case the maximum likelihood estimates for the elapsed times of recent homogenization events at the loci are much older than 200,000 yr B.P. (Table 2). Thus, these estimates disagree with the “Out of Africa” hypothesis.

**Table 1:** Summary of the DNA sequence data sets from loci 22q11.2, 17q23, Xq13.3, and YAP.

Locus	seq. length 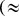 kb)^a^	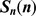 b African	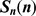 Non-African	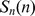 Combined
22q11.2	10	54(40)	44(88)	75(128)
17q23	20	57(10)	37(12)	63(22)
Xq13.3	10	24(23)	17(46)	33(69)
YAP	2.6	3(8)	1(7)	3(15)

a Sequence length in kilobases.

b The number of polymorphic sites in a sample of 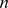 sequences.

**Table 2:** Estimates for the elapsed times 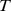 since a recent homogenization event for each of the four loci.

Loci	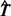 a 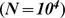	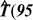 % 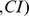 b 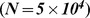 African	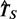 c 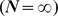	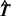 a 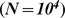	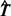 (95 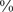 CI)^b^ 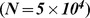 Non-African	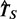 c 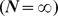
22q11.2	1400	220(120, 380)	117	640	72 (44, 116)	43
17q23	800	320 (220, 500)	247	360	160 (105, 260)	134
Xq13.3	525	127 (71, 240)	90	120	41 (22, 75)	32
YAP	350	195(40, 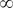 )	125	94	55(0, 450)	30
Combined	1300	241(183, 316)	145	360	92 (67, 125)	55

a The estimates of the elapsed times are in 1000 years Before Present. The estimate of 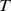 are based on formula (13) when 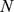 is equal to 10000.

b The estimate of 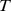 based on formula (13) when 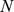 is equal to 50000.

c Under the “simple” model an estimator for 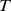 is denoted as 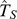. It is equal to 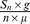 based on the data at a single locus; for data sets from 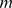 independent loci, it is equal to 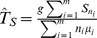.

To explore another possibility, I also consider human effective population size 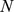 to be 50000 based on the following observations: (1) Some studies [38]–[40] estimated effective human population size to be a few times larger than 10000. (2) Maximums of the likelihood functions of the data sets favor the case 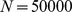 over the case 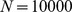 for all the data sets. Thus, I consider the values of 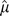 and 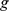 to be the same as above but 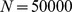. After applying Test I to the data sets from each locus, the likelihood-ratio tests rejected the null hypotheses at 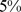 significance level, the results are in Table 3. Clearly, the results suggest that the standard coalescent framework is inadequate to describe the data sets for this set of parameter values, and recent homogenization events have impact on the four loci. The maximum likelihood estimates (see Table 2) of the elapsed times agree with the times for the two major events.

**Table 3:** The values of minus twice of the log of likelihood-ratio statistics for the data sets from each of the four loci.

Locus	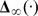 ( 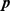 -value) African	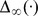 ( 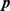 -value) Non-African
22q11.2	19.8 (8.5e−6)	48.6 (0.3e−11)
17q23	11.2 (0.8e−3)	19.8 (0.8e−5)
Xq13.3	20.3 (7e−6)	46.6 (0.8e−11)
YAP	2(0.16)	4.6(0.03)

In this case, the likelihood functions of the data sets would not change dramatically as 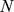 gets larger than 50000 because they behave in large 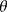 regime. The maximum likelihood estimates of 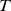, when 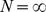, are in Table 2. These estimates show that considering the human effective population size greater than 50,000, the estimates for the elapsed times would not change dramatically.

For this set of parameter values, I use Test III to differentiate the recent homogenization events at the four loci. The results of the tests are in Table 4. These results suggest that the four loci have not been affected by the same homogenization event, 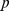-values are less than 0.05 for the data sets from African and Non-African populations. The locus Xq13.3 is significantly younger than the locus 17q23, in particular for Non-African population, which suggest that the locus Xq13.3 has been affected by a recent positive selection or a recent bottleneck occurred to Non-African female population. Using Tajima's test [41] and Fu's and Li's tests [42], Zhao at el. [31] also observed that the diversity at locus Xq13.3 significantly deviates from the Wright-Fisher neutral model.

**Table 4:** The values of minus twice of log of likelihood-ratio statistics for Test III.

Compared loci^a^	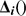 ( 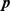 -value) African	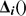 ( 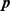 -value) Non-African
(22q11.2, Xq13.3)	1.7 (0.19)	2 (0.15)
(17q23, 22q11.2)	1.3 (0.25)	5.6 (0.02)
(Xq13.3, 17q23)	5.8 (0.02)	12.5 (0.0004)
(YAP, 17q23)	0.3 (0.6)	0.9 (0.3)
(YAP, Xq13.3)	0.2 (0.65)	0.08 (0.8)
(YAP, 22q11.2)	0.01 (0.9)	0.06 (0.8)
(Xq13.3, 17q23, 22q11.2)	6 (0.05)	14 (0.001)
(YAP, Xq13.3, 17q23, 22q11.2)	53 (1.8e−11)	14 (0.003)

a Sets of compared loci.

### Application of the method for inferring the times of HIV seroconversions in HIV-1-infected patients

Usually, after few weeks of HIV infection, plasma viraemia in infected patient declines rapidly as a result of a primary immune response, which coincides with HIV seroconversion [43], [44]. In particular, HIV envelop gene at this time point shows no diversity [45]. To examine the utilities of the framework developed in this paper, I use DNA sequence data from HIV-1 envelop genes published in [46] to infer the times of HIV seroconversions in nine HIV-1-infected patients. The sequences are sampled from the patients at the first HIV-positive screening tests. The sequences are 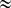
650 nucleotide long; a summary of the data is in Table 5.

**Table 5:** Summary of Shankarappa et al's [46] data.

Patient number^a^	seroconverion time (in years)^b^	sample size^c^	number of polymorphic sites^d^	viral load^e^
1	0.28	7	7	6637
2	0.42	21	33	68706
3	0.35	10	9	598
5	0.25	22	41	7798
6	0.21	19	21	4709
7	0.2	19	32	6251
8	0.29	7	5	4045
9	0.25	10	31	145545
11	0.21	8	13	478

a I used the same notation for the patients as in [46].

b In [46] seroconversion times in the patients are estimated as the mid-time point between the last HIV negative screening test and the first HIV positive screening test.

c For each patient, the samples of DNA sequences are drawn from HIV populations in HIV patients at the time of the first HIV positive screening test.

d The number of polymorphic sites in the samples.

e For each patient viral load per milliliter is measured at the time of the first HIV positive screening test.

For consistency of the data sets with the infinite-sites mutation model and with no intra-locus recombination, the following conditions are checked: (a) Each polymorphic site is a result of a single mutation event, that is only two nucleotide states are possible at each polymorphic site. (b) All pairs of sites in sample of DNA sequences pass the four-gamete test [47]–[49]. Seven of the nine data sets (except data sets from patients 2 and 5) satisfy conditions (a) and (b). The data sets from patients 2 and 5 are inconsistent with the conditions (a) and (b), respectively. However, the two data sets are not excluded from the analysis because inconsistencies in these data sets are a result of two mutations and some recombination events, respectively.

I consider the following values for the parameters in the model: population size 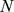 equal to the viral load at the sampling time point, mutation rate 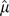 per nucleotide site per generation equal to 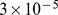, the number of nucleotides at the locus 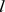 is equal to 650. All the insertions and deletions are excluded from the analysis. For this set of parameter values, I applied Test I to the data from each patient; the null hypotheses are rejected at 5% significance level in favor of recent homogenisation events. For each patient the maximum likelihood and 95% confidence interval estimates of 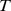 (in coalescent units) are in Table 6. These estimates can be converted in years by using the equation 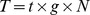, in which the effective HIV generation time 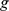 is considered to be equal to 1 or 2 days [50]–[52].

**Table 6:** The estimates of the seroconversion times (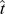 in coalescence units) in the nine patients.

Patient	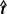 a (95 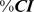 )	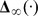	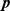 -value
1	0.008 (0.003, 0.016)	36.6	1.2e−9
2	0.0012 (0.0008, 0.0017)	162.1	0
3	0.093 (0.04, 0.21)	13.4	2.5e−4
5	0.013 (0.009, 0.018)	75.3	0
6	0.013 (0.008, 0.02)	65.6	5.5e−16
7	0.015 (0.01, 0.02)	64.4	9.9e−16
8	0.009 (0.003, 0.02)	33.5	7.1e−9
9	0.001 (0.00075, 0.0015)	88.9	0
11	0.23 (0.08, 0.58)	6.2	0.012

a The maximum likelihood estimates of 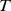 in coalescent units.

**Figure 2:**
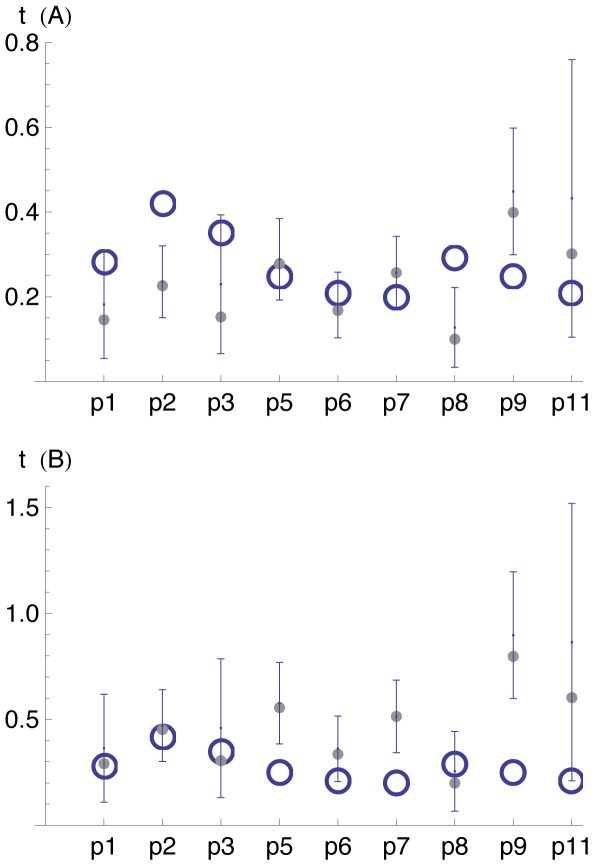
Comparison of two estimates of the seroconversion time for each of the nine patients. The effective generation time 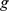 in (A) and (B) are considered to be 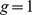 day and 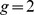 days, respectively. Maximum likelihood and 95% confidence interval estimates of the time of HIV seroconversion in years since the first HIV positive screening test are shown in full dots and error bars, respectively. Empty circles represent the mid-point estimates of the seroconversion times [46].

These estimates are contrasted with the estimates provided by Shankarappa et. al [46]; they estimated the time of HIV seroconversion for each of the patients as the mid-time point between the last HIV-negative and first HIV-positive screening tests. The comparison between the estimates (see Figure 2) shows that for some of the data sets the estimates are significantly in disagreement.

The observed disagreements are robust with respect to 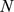 (data not shown): when 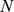 is larger than the viral load, the likelihood functions do not change because of large 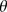 regime. I have also applied the above estimation method by considering 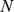 equal to the one tenth of the viral loads. The result show that the observed discrepancies also hold for this case. Note that the viral load represents approximately 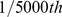 of the total amount of the virus in an HIV-infected person since there is a total of 5 liters of blood in the body of an average adult.

## Discussion

The analytical method developed in this paper is a trade-off between computational efficiency and complexity of the underlying evolutionary model. Using multi-locus DNA sequence data, the method allows identification and differentiation of the signatures of recent severe bottleneck and hitchhiking effects in a computationally efficient way. However, the method uses the number of polymorphic sites instead of full polymorphism data in samples of DNA sequences, and it is constrained by the assumptions of the constant size Wright-Fisher reproduction model and the infinite-sites model. In contrast, coalescent based simulation methods can be implemented at the cost of computational feasibility to include full polymorphism data [7], various demographic scenarios [6], and finite-sites mutation models [53]. However, before using computationally more expensive methods, the method could be a helpful guide for analyzing multi-locus DNA sequences data.

**Figure 3:**
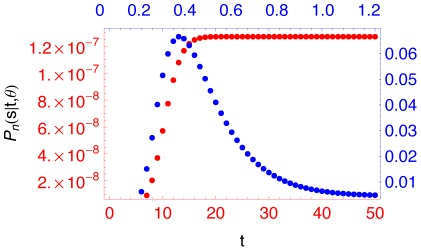
The likelihood function of 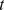
 **for two values of** 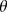 **.** For a sample of 15 DNA sequences with 25 polymorphic sites at a locus, the likelihood function of the elapsed time 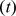 is plotted for the values of 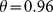 and 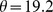 in red and blue, respectively.

To illustrate the behavior of the likelihood function for small and large 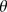, I used the program to plot the likelihood function of 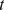 (see Figure 3) for a sample of 15 DNA sequences with 25 polymorphic sites when 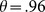 and 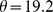. The behavior of the likelihood function can be explained based on the process that traces ancestral lineages of the sample back in time. When tracing 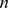 lineages back in time, coalescent and mutation events occur one at a time with rates 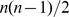 and 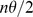, respectively. Thus, when 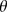 is large, mutation events occur more often than coalescent events back in time, so for a given number of polymorphic sites the recent homogenization event is more likely to be before the most recent common ancestor of the sample. This also explains the approximation (14). In opposite to this, when 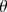 is small, the sample is more likely to have the most recent common ancestor before the recent homogenization event. Similarly, as 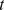 gets larger the sample is more likely to have the most recent common ancestor before the homogenization event, hence the likelihood function has a limit (see (15)).

Computational efficiency of the method gives an advantage to explore various values for the parameters in the model for assessing the impact of parameter values on the inference. The application of the method to the human data shows that when the effective human population size 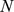 is equal to 10000 or greater than 50000, the inferences about evolutionary history of modern human populations are dramatically different. The HIV data analysis shows that the observed discrepancies between estimates for HIV seroconversions in the patients can be a result of the assumption that the effective HIV generation time is the same for all the patients. To have a better assessment for this assumption, frequent HIV screening tests can be used to assess the times of HIV seroconversion in HIV patients, and then to apply this method for exploring variability of effective HIV-1 generation times between HIV patients.

As the analysis of the human DNA sequences data shows the method developed in this paper does not have enough power to give an estimate for the effective human population size. Although the method suggest that very large values of 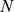 as maximum likelihood estimates for some of the human data sets when 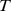 and 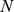 are considered unknown, this does not mean that the “simple” model (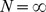) is an appropriate model for explaining the data sets because site frequency spectrum of a sample of DNA sequences under the simple model consists only singletons, and Zhao et al. [31] observed excess number of singletons and doubletons for all the data sets. Note that under the model considered in this paper the behavior of the expected site frequency spectrum in samples of DNA sequences changes continuously respect to 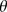, for example when the effective population size 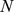 changes continuously. The two extreme ends of the expected site frequency spectrum under this model are described by the standard coalescent and by the “simple” model, respectively for small and very large values of 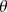. Under the standard coalescent the expected site frequency spectrum represents a wide range for frequencies of alleles. Thus, as 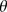 (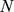) increases the expected number of low-frequency alleles increases.

## Supporting Information

Text S1 (PDF)
