# Supplementary material for: Analytical Framework for Identifying and Differentiating Recent Hitchhiking and Severe Bottleneck Effects from Multi-Locus DNA Sequence Data
Source: PLoS One. 2012 May 25;7(5):e37588. doi: 10.1371/journal.pone.0037588 (PMC3360760; doi:10.1371/journal.pone.0037588)
Supplement: Text S1 — (PDF) [file pone.0037588.s001.pdf]

Supporting Information

The proof of Lemma 1

Griffiths [1] showed that $g_n(u,t)$ satisfies the recurrence relation:

$$g_n(u,t) = \frac{n(n-1)}{2}\int_0^t \phi_n(u,t-s)g_{n-1}(u,s)ds + \phi_n(u,t), \tag{1}$$

with initial condition $g_1(u,t) = 1$, where $\phi_n(u,s) = e^{-ns(2u+n-1)/2}$. Because the solution of the recurrence relation is unique, to prove Lemma 1, we need to be shown that the function $f_n(u,t)$ defined by the expression

$$f_n(u,t) \equiv \phi_n(u,t) + \binom{n}{2}\sum_{i=2}^{n}\sum_{\substack{j=0\\ j\neq 1}}^{i-1}\frac{\mu_{i,j}^{(n)}(i+j-1)}{-(u+\frac{i+j-1}{2})}\xi(i,j,t,u)$$

satisfies the recurrence relation (1). In other words, the equality

$$f^*_{k+1}(u,t) = f_{k+1}(u,t), \tag{2}$$

needs to be shown, where $f^*_{n+1}(u,t)$ is defined as

$$f^*_{n+1}(u,t) \equiv \binom{n+1}{2}\int_0^t \phi_{n+1}(u,t-s)f_n(u,s)ds + \phi_{n+1}(u,t), \tag{3}$$

for any integer k. To prove this, mathematical induction with respect to k is used. That is, first the equalities $f_1(u,t) = 1$ and $f^*_2(u,t) = f_2(u,t)$, are checked, for $k = 1$; second, assuming that the equation (2) holds for $k = 1,\dots,n-1$, below I show that the equation also holds for $k = n$. To show this, $f_n(u,t)$ in the right side of the equation (3) is substituted by its expression:

$$f^*_{n+1}(u,t) = \phi_{n+1}(u,t) + \binom{n+1}{2}\int_0^t \phi_{n+1}(u,t-s)\phi_n(u,s)ds \tag{4}$$

$$+\binom{n+1}{2}\binom{n}{2}\sum_{i=2}^{n}\sum_{\substack{j=0\\ j\neq 1}}^{i-1}\frac{\mu_{i,j}^{(n)}(i+j-1)}{-(u+\frac{i+j-1}{2})}\int_0^t \phi_{n+1}(u,t-s)\xi(i,j,s,u)ds$$

The integrals in the above equation are simplified by using the following formula

$$\int_0^t \phi_b(u,t-s)\exp(-sa(u+(a-1)/2))ds = \frac{\xi(a,b,t,u)}{-(a-b)(u+\frac{a+b-1}{2})}.$$

Thus, the integral terms can be represented as

$$\frac{\mu_{i,j}^{(n)}(i+j-1)}{-(u+\frac{i+j-1}{2})}\left(\frac{\xi(i,n+1,t,u)}{(n+1-i)(u+\frac{n+i}{2})}-\frac{\xi(j,n+1,t,u)}{(n+1-j)(u+\frac{n+j}{2})}\right)$$

and

$$\int_0^t \phi_{n+1}(u,t-s)\phi_n(u,s)ds=\frac{\xi(n,n+1,t,u)}{(u+n)}.$$

After using the above simplifications with the identities

$$\frac{1}{\left(u+\frac{i+j-1}{2}\right)\left(u+\frac{n+i}{2}\right)}=\frac{2}{n+1-j}\left(\frac{1}{u+\frac{i+j-1}{2}}-\frac{1}{u+\frac{n+i}{2}}\right)$$

and

$$\frac{1}{\left(u+\frac{i+j-1}{2}\right)\left(u+\frac{n+j}{2}\right)}=\frac{2}{n+1-i}\left(\frac{1}{u+\frac{i+j-1}{2}}-\frac{1}{u+\frac{n+j}{2}}\right),$$

in the right side of (4), the following equation holds

$$\begin{aligned}
f_{n+1}^*(u,t) &= \phi_{n+1}(u,t)+\binom{n+1}{2}\frac{\xi(n,n+1,t,u)}{(u+n)} \qquad (5)\\
&+ \binom{n+1}{2}\sum_{i=2}^{n}\sum_{\substack{j=0\\ j\neq 1}}^{i-1}\binom{n}{2}\frac{2\mu_{i,j}^{(n)}(i+j-1)}{-(n+1-i)(n+1-j)}\frac{\xi(i,j,t,u)}{(u+\frac{i+j-1}{2})}\\
&- \binom{n+1}{2}\sum_{i=2}^{n}\sum_{\substack{j=0\\ j\neq 1}}^{i-1}\binom{n}{2}\frac{2\mu_{i,j}^{(n)}(i+j-1)}{-(n+1-i)(n+1-j)}\frac{\xi(i,n+1,t,u)}{(u+\frac{n+i}{2})}\\
&+ \binom{n+1}{2}\sum_{i=2}^{n}\sum_{\substack{j=0\\ j\neq 1}}^{i-1}\binom{n}{2}\frac{2\mu_{i,j}^{(n)}(i+j-1)}{-(n+1-i)(n+1-j)}\frac{\xi(j,n+1,t,u)}{(u+\frac{n+j}{2})}.
\end{aligned}$$

Recall that the coefficients $\{\mu_{i,j}^{(n)}\}$ are determined by the following recurrence relations

with initial conditions:

$$\mu_{i,j}^{(n)} = \frac{(n-1)(n-2)}{(n-j)(n-i)}\mu_{i,j}^{(n-1)},\ 2 \le i \le n-1, j=0,\ \text{or } 2 \le j \le i-1; \tag{6}$$

$$\mu_{n,k}^{(n)} = \sum_{\substack{j=0 \\ j\neq 1}}^{k-1} \mu_{k,j}^{(n)} - \sum_{i=k+1}^{n-1} \mu_{i,k}^{(n)},\quad 2 \le k \le n-1; \tag{7}$$

$$\mu_{2,0}^{(2)} = \frac{1}{2}, \tag{8}$$

$$\mu_{n,0}^{(n)} = \frac{(-1)^n}{n(n-1)}. \tag{9}$$

and satisfy the following identities

$$\frac{1}{n(n-1)} = \sum_{\substack{j=0 \\ j\neq 1}}^{n-1} \mu_{n,j}^{(n)}; \tag{10}$$

$$\frac{1}{n(n-1)} = \sum_{i=2}^{n} \mu_{i,0}^{(n)}; \tag{11}$$

$$\mu_{i,0}^{(n)} = \frac{(-1)^i \binom{n-2}{i-2}}{n(i-1)}. \tag{12}$$

The equation (5) can be presented as

$$f_{n+1}^{*}(u,t) = \phi_{n+1}(u,t) + \binom{n+1}{2}\frac{\xi(n+1,n,t,u)}{-(u+n)}$$

$$+\binom{n+1}{2}\sum_{i=2}^{n}\sum_{\substack{j=0 \\ j\neq 1}}^{i-1}\frac{\mu_{i,j}^{(n+1)}(i+j-1)\xi(i,j,t,u)}{-(u+\frac{i+j-1}{2})}$$

$$-\binom{n+1}{2}\sum_{i=2}^{n}\sum_{\substack{j=0 \\ j\neq 1}}^{i-1}\frac{\mu_{i,j}^{(n+1)}(i+j-1)\xi(i,n+1,t,u)}{-(u+\frac{n+i}{2})}$$

$$+\binom{n+1}{2}\sum_{i=2}^{n}\sum_{\substack{j=0 \\ j\neq 1}}^{i-1}\frac{\mu_{i,j}^{(n+1)}(i+j-1)\xi(j,n+1,t,u)}{(u+\frac{n+j}{2})}$$

by using (6), that is

$$\binom{n}{2}\frac{2\mu_{i,j}^{(n)}}{(n+1-i)(n+1-j)} = \mu_{i,j}^{(n+1)},\ 2 \le i \le n, j = 0,\ \text{or}\ 2 \le j \le i-1.$$

After modifying the expressions

$$-\binom{n+1}{2}\sum_{i=2}^{n}\sum_{\substack{j=0\\ j\neq 1}}^{i-1}\frac{\mu_{i,j}^{(n+1)}(i+j-1)\xi(i,n+1,t,u)}{-(u+\frac{n+i}{2})}$$

and

$$+\binom{n+1}{2}\sum_{i=2}^{n}\sum_{\substack{j=0\\ j\neq 1}}^{i-1}\frac{\mu_{i,j}^{(n+1)}(i+j-1)\xi(j,n+1,t,u)}{-(u+\frac{n+j}{2})}$$

in the above equation respectively by

$$\binom{n+1}{2}\sum_{i=2}^{n}\frac{\xi(n+1,i,t,u)}{-(u+\frac{n+i}{2})}\sum_{\substack{j=0\\ j\neq 1}}^{i-1}\mu_{i,j}^{(n+1)}(i+j-1)$$

and

$$-\binom{n+1}{2}\sum_{\substack{j=0\\ j\neq 1}}^{n-1}\frac{\xi(n+1,j,t,u)}{-(u+\frac{n+j}{2})}\sum_{\substack{i=j+1\\ i=2\ \text{if}\ j=0}}^{n}\mu_{i,j}^{(n+1)}(i+j-1),$$

and taking into account the definition of $f_{n+1}(u,t)$, it is easy to see that the the equality $f_{n+1}^*(u,t) = f_{n+1}(u,t)$ holds if the following equations hold:

$$1+\sum_{\substack{j=0\\ j\neq 1}}^{n-1}\mu_{n,j}^{(n+1)}(n+j-1) = 2n\mu_{n+1,n}^{(n+1)}, \tag{13}$$

$$-\sum_{i=2}^{n}\mu_{i,0}^{(n+1)}(i-1) = n\mu_{n+1,0}^{(n+1)}, \tag{14}$$

and

$$\sum_{\substack{k=0\\ k\neq 1}}^{i-1}\mu_{i,k}^{(n+1)}(i+k-1) - \sum_{k=i+1}^{n}\mu_{k,i}^{(n+1)}(k+i-1) = (n+i)\mu_{n+1,i}^{(n+1)},\ i = 2,\ldots,n-1. \tag{15}$$

To prove equation (13), first the left side of the equation is modified using (7) , (6), and (10) . As a result, the following equalities hold:

$$1+\sum_{\substack{j=0\\j\neq 1}}^{n-1}\mu_{n,j}^{(n+1)}(n+j-1)=\sum_{\substack{j=0\\j\neq 1}}^{n-1}\mu_{n,j}^{(n+1)}(2n)+1-\sum_{\substack{j=0\\j\neq 1}}^{n-1}\mu_{n,j}^{(n+1)}(n+1-j)$$

$$=2n\mu_{n+1,n}^{(n+1)}+1-\sum_{\substack{j=0\\j\neq 1}}^{n-1}\mu_{n,j}^{(n)}n(n-1)=2n\mu_{n+1,n}^{(n+1)}.$$

The proof of the equation (14) follows after applying (12) to the left side of (14):

$$-\sum_{i=2}^{n}\mu_{i,0}^{(n+1)}(i-1)=-\sum_{i=2}^{n}\frac{(-1)^i\binom{n-1}{i-2}}{(n+1)(i-1)}(i-1)=-\frac{1}{n+1}\sum_{i=0}^{n-2}(-1)^i\binom{n-1}{i}$$

$$=\frac{1}{n+1}(-1)^{n-1}\binom{n-1}{n-1}=\frac{(-1)^{n+1}}{n+1}=n\mu_{n+1,0}^{(n+1)}.$$

To prove (15), the left side of the equation is modified by using (7) and (6). That is,

$$\sum_{\substack{k=0\\k\neq 1}}^{i-1}\mu_{i,k}^{(n+1)}(i+k-1)-\sum_{k=i+1}^{n}\mu_{k,i}^{(n+1)}(k+i-1)=(n+i)\left(\sum_{\substack{k=0\\k\neq 1}}^{i-1}\mu_{i,k}^{(n+1)}-\sum_{k=i+1}^{n}\mu_{k,i}^{(n+1)}\right)$$

$$+\left(-\sum_{\substack{k=0\\k\neq 1}}^{i-1}\mu_{i,k}^{(n+1)}(n+1-k)+\sum_{k=i+1}^{n}\mu_{k,i}^{(n+1)}(n+1-k)\right)$$

$$=(n+i)\mu_{n+1,i}^{(n+1)}+\left(-\sum_{\substack{k=0\\k\neq 1}}^{i-1}\mu_{i,k}^{(n)}\frac{n(n-1)}{n+1-i}+\sum_{k=i+1}^{n}\mu_{k,i}^{(n)}\frac{n(n-1)}{n+1-i}\right)=(n+i)\mu_{n+1,i}^{(n+1)}.$$

The proof of Lemma 1 is complete.

An expression for the density function of the total length of the genealogy of a sample

After inverting the expression the following lemma holds for the density function of $L_n(t)$.

Lemma *The random variable $L_n(t)$ has density function given by the formula*

$$\binom{n}{2}\sum_{i=2}^{n}\sum_{\substack{j=0\\ j\neq 1}}^{i-1}\mu_{i,j}^{(n)}(i+j-1)\exp\left(\frac{ijt-(i+j-1)s}{2}\right)\mathbf{I}(jt,it),$$

when $0<s<nt$ *and a point mass* $\exp(-n(n-1)t/2)$ *at* nt*. Here* $\mathbf{I}(a,b)$ *is the indicator function on the interval* (a,b)*.* The prove of the formula easily follows from Lemma 1 and the uniqueness of Laplace transformation.

Acknowledgments

References

1. Griffiths R (1981) Transient distribution of the number of segregating sites in a neutral infinite-sites model with no recombination. J Appl Prob 18: 42 - 51.
